# A Fetus with Imperforate Anus Developing Pulmonary Hypoplasia Triggered by Transient Urethral Obstruction

**DOI:** 10.1155/2021/9950578

**Published:** 2021-05-13

**Authors:** Masatake Toshimitsu, Takayuki Iriyama, Seisuke Sayama, Kan Suzuki, Satsuki Kakiuchi, Mari Ichinose, Takahiro Seyama, Kenbun Sone, Keiichi Kumasawa, Takeshi Nagamatsu, Tomoyuki Fujii, Yutaka Osuga

**Affiliations:** ^1^Department of Obstetrics and Gynecology, Faculty of Medicine, The University of Tokyo, 7-3-1 Hongo, Bunkyo-ku, Tokyo 113-8655, Japan; ^2^Department of Pediatric Surgery, Faculty of Medicine, The University of Tokyo, 7-3-1 Hongo, Bunkyo-ku, Tokyo 113-8655, Japan; ^3^Department of Pediatrics, Faculty of Medicine, The University of Tokyo, 7-3-1 Hongo, Bunkyo-ku, Tokyo 113-8655, Japan; ^4^Department of Obstetrics and Gynecology, Sanno Hospital, 8-10-16 Akasaka, Minato-ku, Tokyo 107-0052, Japan

## Abstract

Pulmonary hypoplasia is a rare entity in a fetus with imperforate anus. The fetus was diagnosed with high-type imperforate anus with rectourethral fistula based on the dilated fetal bowel and the presence of bowel calcification at 19 weeks of gestation. As gestation advanced, fetal ultrasonography demonstrated development of pulmonary hypoplasia, progressive bowel dilation, and persistent oligohydramnios from 28 weeks of gestation despite a fluid-filled bladder without hydroureter or hydronephrosis. To prevent further worsening of pulmonary hypoplasia caused by thoracic compression due to bowel dilation and oligohydramnios, a male neonate was delivered by cesarean section at 32 weeks of gestation. The neonate showed respiratory failure requiring full respiratory support. Although a catheter did not pass through the urethra into the bladder at birth, cystourethrography revealed the patency of fistula and stenosed lower urinary tract. Prenatal and postnatal findings strongly suggested that the meconium in the colon might have passed into the urethra in the penis, resulting in the physical blockage of urine outflow to the amniotic space which leads urine flow from the bladder to the colon through the fistula, which resulted in subsequent oligohydramnios and bowel dilation. To the best of our knowledge, this is the first case report of a fetus with imperforate anus developing pulmonary hypoplasia possibly due to urethral obstruction.

## 1. Introduction

Imperforate anus is a relatively common congenital anomaly and is prenatally diagnosed by fetal ultrasonography based on direct signs, such as the absence of fetal anal mucosa and sphincter muscle [1]. The prognosis of a fetus with imperforate anus is dependent on the location of the rectal end, categorized as low, intermediate, and high type, and the presence of other congenital complex such as VACTERL association or aneuploidy such as trisomy 21 [2]. Among them, intermediate/high type has rectourethral fistula that can result in intrabowel mixing of meconium and urine leading to fetal bowel calcification in the dilated bowel [3, 4]. Reciprocally, the presence of fetal bowel calcification implies that there may be rectourethral fistula and indicates intermediate-/high-type imperforate anus.

Despite recent progress in neonatal management, pulmonary hypoplasia is lethal and still the most important cause of neonatal death [5]. Pulmonary hypoplasia can be caused by multiple factors such as reduction in amniotic fluid, intrathoracic cavity size, or fetal breathing movement [6]. However, there has never been any report showing a possible causal relationship between imperforate anus and pulmonary hypoplasia.

Here, we present a case of an isolated imperforate anus diagnosed prenatally that developed pulmonary hypoplasia caused by progressive bowel dilation and oligohydramnios, in which the urine flow was directed from the bladder to the colon instead of the amniotic space through the rectourethral fistula due to urethral obstruction.

## 2. Case Report

**Figure 1:**
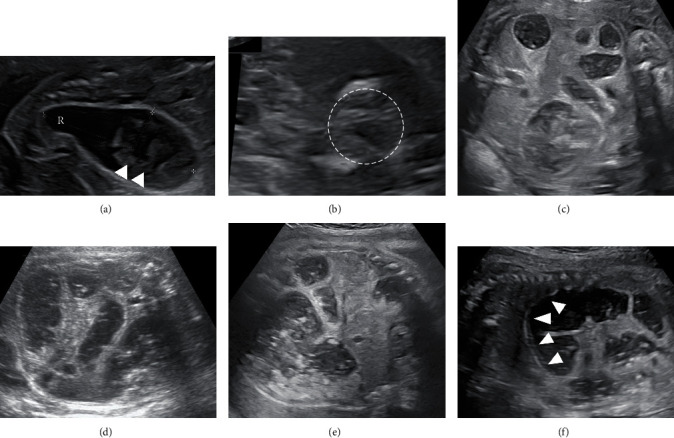
Fetal ultrasonography and the change of fetal bowel. Fetal images. (a) The distal rectal pouch shows dilated bowel with intraluminal calcifications (arrowheads) at 20 weeks. R indicates distal rectal pouch. The arrowheads indicate bowel calcifications. (b) The echogenic center (dotted circle) is not detectable at 20 weeks, which indicates that anal mucosa is invisible. (c) Fetal bowel dilation at 26 weeks. (d) Fetal bowel dilation at 31 weeks. (e) Fetal bowel dilation at 32 weeks. (f) Thoracic compression by bowel dilation at 31 weeks. The arrowheads indicate the diaphragm.

**Figure 2:**
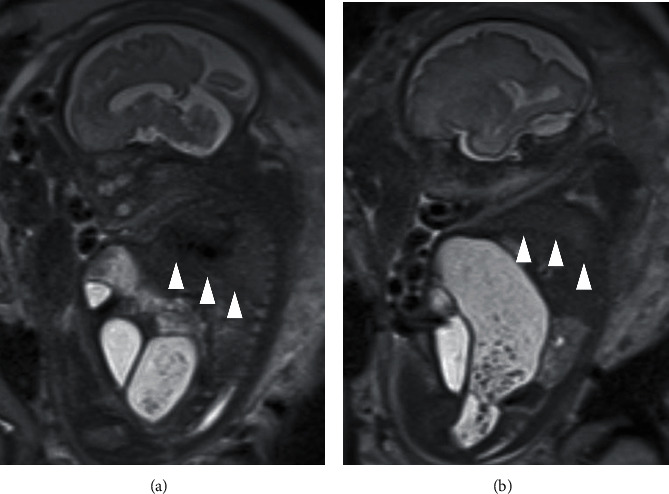
Fetal MRI. Fetal T2-weighted magnetic resonance imaging at 30 weeks showing small lungs with bowel dilation and oligohydramnios. The arrowheads indicate the diaphragm.

**Figure 3:**
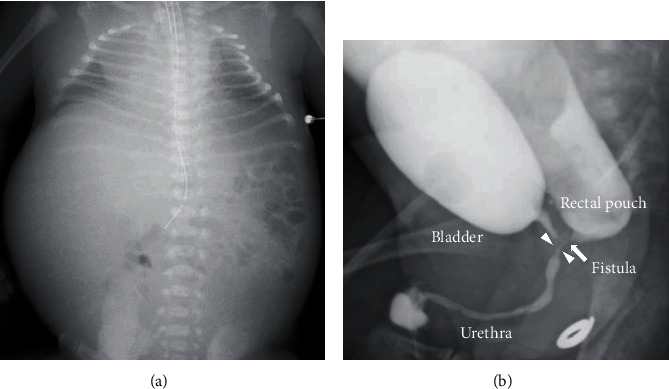
Neonatal chest X-ray and cystourethrography: (a) chest X-ray on day 1 showing poor lung expansion and abdominal expansion; (b) cystourethrography showing a patent rectourethral prostatic fistula into the rectal pouch and a patent urethra. The arrowheads indicate urethral stenosis distal to rectourethral fistula. The arrow indicates rectourethral prostatic fistula.

A 30-year-old nulliparous pregnant Japanese woman was referred to our institution for prenatal evaluation of a fetal abdominal cyst at 19 weeks of gestation. Fetal ultrasonography showing a distended colon with the presence of bowel calcifications, distal rectal pouch, and undetectable anal mucosa indicated the presence of intermediate- or high-type imperforate anus with rectourethral fistula (Figures 1(a) and 1(b)). No other anomaly or fetal growth restriction was detected (Table 1). Fetal ultrasonography at 20 weeks of gestation showed the progression in colon dilation and relatively decreased amniotic fluid but not yet oligohydramnios. Even after informing the parents of possible poor prognosis of the fetus, the parents did not desire termination of pregnancy or amniocentesis. Fetal ultrasonography showed further progression of bowel dilation obviously seen from 26 weeks of gestation, which leads to thoracic compression, and amniotic fluid suddenly decreased after 28 weeks of gestation despite a fluid-filled bladder without hydroureter or hydronephrosis, which implied the presence of fetal urine outflow obstruction but not a decrease in urine production (Figures 1(c)–1(f), Table 1). Sonographic assessment of fetal well-being, including Doppler velocimetry of the middle cerebral artery, umbilical cord artery, and ductus venous, and biophysical profiling, was normal. MRI was performed at 30 weeks of gestation to assess pulmonary development, which indicated possible pulmonary hypoplasia (Figure 2). By 32 weeks of gestation, although the fetal bladder was filled, oligohydramnios persisted without any signs of premature rupture of the membranes, placental insufficiency, hydroureter, or hydronephrosis. Taken together with the progression of bowel dilation which distended the fetal abdomen, fetal urethral stenosis or obstruction was expected to have triggered a misdirected urine flow from the bladder to the colon through the fistula. As deterioration of oligohydramnios and bowel dilation which lead to pulmonary hypoplasia by thoracic compression was progressive, expectant management was anticipated to worsen the prognosis of the fetus. Therefore, antenatal corticosteroid therapy for lung maturation was administered and the male neonate was delivered by cesarean section at 32 weeks and 5 days of gestation. Birth weight was 2473 g with an Apgar score of 5 at 1 min and 7 at 5 min. The umbilical artery showed pH 7.30, PaCO_2_ 45.8, PO_2_ 24.1, and BE -3.9. Physical examination of the neonate confirmed imperforate anus and a remarkable distended abdomen. The neonate required immediate intubation and high-frequency oscillatory ventilation accompanied with inhaled nitric oxide treatment due to severe respiratory failure with persistent pulmonary hypertension (Figure 3(a)). Regarding imperforate anus, cystostomy was performed on the first day, because a 3 Fr catheter did not pass through urethra into the fluid-filled bladder and abdominal ultrasonography showed a stone-like hyperechoic object in the urethra. On 36 days after birth, cystourethrography revealed high-type imperforate anus with patent rectourethral fistula, nonobstructive lower urinary tract, and urethral stenosis distal to the rectourethral fistula (Figure 3(b)). Postnatal MRI did not demonstrate any cerebral abnormalities including periventricular leukomalacia or intraventricular hemorrhage. The neonate was discharged after posterior sagittal anorectoplasty without respiratory complication and is doing well at 19 months after birth.

## 3. Discussion

This report describes the first case of a characteristic clinical course of high-type isolated imperforate anus with transient urethral obstruction causing pulmonary hypoplasia. Urethral obstruction directed the urine flow from the bladder through the rectourethral fistula to the colon, causing oligohydramnios, progression of bowel dilation, and pulmonary hypoplasia requiring termination of pregnancy to treat the fetus outside of the uterus and give respiratory support after birth.

Prenatal diagnosis of imperforate anus is important as neonatal management differs depending on imperforate anus subtype and the presence of other congenital anomalies such as urological abnormalities [2, 7]. However, it has been challenging to evaluate the type of imperforate anus and additional anomalies in detail prenatally [1]. In the present case, as previously reported [3, 4], intermediate or high imperforate anus was diagnosed based on the presence of bowel calcifications in the distended colon at 19 weeks of gestation.

**Figure 4:**
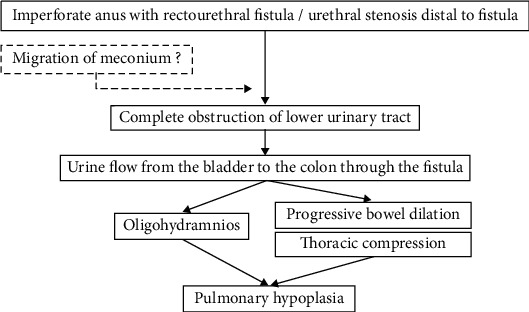
Schematic of the proposed mechanism of pulmonary hypoplasia in our case. The narrow urethra may have been obstructed due to fetal meconium, which may have passed from the colon into the urethra through the fistula, resulting in complete obstruction of the lower urinary tract in the fetus with urethral stenosis. Urethral obstruction directed the urine flow from the bladder through the rectourethral fistula to the colon, causing oligohydramnios and progression of bowel dilation, resulting in pulmonary hypoplasia.

One of the characteristic findings in our case was the progression of bowel dilation with a sudden onset of oligohydramnios after 28 weeks of gestation and absence of hydroureter or hydronephrosis despite a normal fluid-filled bladder. Postnatal examination did not identify congenital lower urinary tract obstruction such as urethral atresia or posterior urethral valves, but sonography after birth showed a stone-like hyperechoic object in the urethra and cystourethrography revealed the patency of rectourethral fistula, nonobstructive lower urinary tract, and the urethral stenosis distal to the rectourethral fistula. These findings suggest transient obstruction of urinary flow to the amniotic space through the urethra and misdirection of urine flow to the colon through the rectourethral fistula. The exact reason why fetal urine did not pass into to the amniotic space suddenly after 28 weeks of gestation is unclear. There is a chance that the narrow urethra may have been obstructed completely due to some physical inducement such as flexion in the urethra, but given these prenatal and postnatal findings, we hypothesized that calcified fetal meconium may have passed from the colon into the urethra through the fistula, resulting in complete obstruction of the lower urinary tract which superimposed on partial obstruction of urinary tract in the fetus with urethral stenosis (Figure 4).

Another feature of our case was pulmonary hypoplasia, which required full respiratory support after birth. It is recognized that the severity of pulmonary hypoplasia is dependent on its timing of onset and underlying cause [6]. In the present case, there are several possibilities that may have caused pulmonary hypoplasia. One possibility is that the progression of bowel dilation may have resulted in the inhibition of fetal breathing movements or thoracic cavity growth by physical thoracic compression. However, it is uncertain how pulmonary hypoplasia occurred since the impact of thoracic compression by bowel dilation may be different from that of congenital diaphragmatic hernia or neuromuscular disorders. Another possibility is that oligohydramnios from 28 weeks of gestation may have been involved in the development of pulmonary hypoplasia. Despite the exact mechanism which remains unknown, severe oligohydramnios is known to disrupt fetal lung development [6]. In addition, oligohydramnios causes fetal deformities and variable fetal heart rate decelerations in cardiotocography [6]. These complications of persistent oligohydramnios may be ameliorated by repetitive amnioinfusion [8–10]. In the present case, expectant management was performed as for the following reasons. First, oligohydramnios appeared from 28 weeks of gestation, which is the end of the canalicular stage [6]. Second, pulmonary hypoplasia was anticipated to be caused by thoracic compression due to bowel dilation rather than oligohydramnios based on fetal ultrasonography and MRI.

The decision on the timing of delivery was carefully made after assessing the benefit of being able to treat pulmonary hypoplasia caused by thoracic compression due to bowel dilation and urinary tract obstruction and the disadvantage of prematurity from preterm delivery which may increase the risk of neonatal morbidity and mortality, particularly due to respiratory distress. In the present case, preterm delivery at 32 weeks of gestation was chosen as for the following reasons. First, the urinary tract obstruction did not resolve spontaneously in utero from 28 weeks to 32 weeks of gestation. Second, subsequent thoracic compression was deteriorated with gestation. Finally, there was a risk of fetal renal damage by lower urinary tract obstruction if urine reflux into the upper urinary tract occurred. As pulmonary hypoplasia associated with imperforate anus is rare and optimal management is lacking in this setting, the decision on when to terminate may differ based on the delivery policy at each individual institution. Our multidisciplinary approach provided beneficial information in managing the fetus to assess the timing of delivery and prenatal counseling during the perinatal period. Although the cause of pulmonary hypoplasia in this case is still uncertain, it is important to recognize that pulmonary hypoplasia can occur in a fetus with imperforate anus.

In conclusion, pulmonary hypoplasia is rare but occurs in a fetus with imperforate anus triggered by urethral obstruction. It is essential to recognize that the presence of a fistula may cause urine outflow blockage with meconium to the amniotic space at the level of the urethra and subsequent pulmonary hypoplasia. When managing a fetus with imperforate anus complicated with a fistula, careful obstetric management is essential, including thorough follow-up on fetal bowel dilation and amniotic fluid volume to assess the possible onset of pulmonary hypoplasia.

## Figures and Tables

**Table 1:** Prenatal findings of the present case.

GA (weeks + days)	19 + 1	20 + 6	22 + 6	24 + 6	26 + 6	28 + 6	29 + 6	32 + 1
EFBW (g)	290	402	666	882	1291	1467	1932	2330
(SD)	(±0)	(-0.2)	(+0.8)	(+0.6)	(+1.5)	(+0.6)	(+2.1)	(+1.8)
AFI (cm)			8.3	7.9	7.1	2.7	1.9	0.6
Bladder	+	+	+	+	+	+	+	+

GA: gestational age; EFBW: estimated fetal body weight; AFI: amniotic fluid index.
